# Climate variability, agricultural technologies adoption, and productivity in rural Nigeria: a plot-level analysis

**DOI:** 10.1186/s40066-023-00411-x

**Published:** 2023-04-13

**Authors:** Akuffo Amankwah

**Affiliations:** Living Standards Measurement Study, Development Data Group, World Bank, Washington, DC USA

**Keywords:** Rural Nigeria, Sustainable agricultural practices, Productivity, Multivariate probit, Plot-level, Climate variability, Q01, Q12, Q16, Q18

## Abstract

**Background:**

Increasing agricultural productivity has long been touted as the main avenue to lifting the rural poor out of poverty and ensuring their sustainable development. The adoption of sustainable agricultural practices (SAPs) is vital for spurring agricultural productivity in a changing climate environment. This paper examines the factors (including long-term climate variability) influencing the adoption of multiple SAPs (improved seeds, organic and inorganic fertilizers, and legume intercropping) and their impact on crop productivity.

**Methodology:**

This study uses a nationally representative, geo-referenced plot-level data from a household survey in Nigeria. A multistage sampling technique was used to select households for the survey. The multivariate and ordered probit models were employed to estimate the adoption and intensity of adoption respectively, while the instrumental variables approach was used to examine the impact of the technologies on productivity.

**Results:**

The results provide evidence of interdependences between the SAPs, and that the factors that determine the initial adoption decisions are not necessarily the same factors that influence the intensity of use of the technologies. Climate risks in terms of high variability in temperature and rainfall affect SAPs adoption and their intensity of use. Access to agricultural extension, years of education and off-farm activities of the plot manager, and household wealth influence the use of improved seeds and inorganic fertilizer. Organic fertilizers are used mainly by households with large livestock units and those that live in areas with low soil nutrients and greenness index. In general, the intensity of SAPs adoption is influenced by wage and off-farm activities, and access to agricultural extension services. On the productivity side, inorganic fertilizer is positively correlated with plot-level productivity.

**Conclusions:**

These results have implications for rural development policies in Nigeria aimed at nudging farmers to adopt multiple technologies on their plots, while enhancing an outward shift of their crop production functions. Providing technical and financial resources to extension agents is crucial so they could better reach rural smallholder households with the knowledge and benefits of these SAPs. In addition, smallholder households should diversify their livelihood portfolios to include non-farm income generating activities. Agricultural Research and Development should target factors that respond to climate variabilities (such as drought resistant and early maturing varieties). There is a need for improved infrastructures (road networks to allow easy market access as well as access to credit) that will allow farmers to access these innovations.

## Introduction

Agriculture continues to be the main source of livelihood of the rural poor in Sub-Saharan Africa (SSA). Increasing agricultural productivity has long been touted as the main avenue to lifting the rural poor out of poverty and ensuring their sustainable development. Agricultural production can be increased by either expanding the area under cultivation, changing cropping systems, and or use of sustainable productivity enhancing practices [41]. Over the years, however, the rise in agricultural production in SSA has emanated mainly from expansion of area under cultivation, with less adoption of sustainable practices [20, 41]. Urbanization and increasing population are, however, causing the conversion of historically agricultural lands into residential. Continuous cropping and land degradation have become rampant, leading to decreased soil fertility, and consequently, low yields and high food insecurity. Variations in climatic conditions—rising temperatures, low and erratic rainfall—continue to pose threats to agriculture and food security, given that agriculture in the sub-region is mainly rainfed [21, 22].

Agriculture is the second largest share of the Nigerian economy, contributing an average of 24% to the country’s Gross Domestic Product (GDP) over the past decade [34]. The growth rate in agriculture decreased from 6.7% in 2012 to 2.13% in 2021. Despite this decline in growth, the sector remains the main source of food, employment, and other forms of livelihood of the rural poor. In 2017, the share of the working population engaged in agriculture was about 60% [35]. According to NBS [32], the share of poor people [those living below the national poverty line of 137,430 Naira (USD 382)^1^ in Nigeria stood at 40.1% in 2020; rural and urban poverty rates were 52.1% and 18%, respectively. The COVID-19 pandemic has exacerbated the food insecurity situation in Nigeria, increasing the share of individuals that are severe food insecure from about 18% in 2019 to more than 30% in 2020 [17]. Thus, ending poverty and food insecurity among the rural poor in Nigeria requires targeting the agriculture sector, including increasing productivity, as well as enhancing access to input, output, and credit markets.

While the government has implemented number of policies aimed at boosting agricultural productivity and food security, rural smallholder households in Nigeria continue to face supply and demand side challenges: notably poor land tenure system, very low level of irrigation development, limited research on agricultural technologies, high cost of farm inputs, poor access to markets, and high postharvest losses and waste [16]. These are further compounded by the negative impacts of changing climate in terms of high temperatures and low and unpredictable rainfall patterns, given that the agriculture sector in Nigeria, like other SSA countries, is mainly rainfed. Increasing agricultural productivity and boosting food security, therefore, requires surmounting these challenges at the plot level, including the adoption of sustainable agricultural practices (SAPs) [49].

There are a number of theoretical and empirical studies on the adoption of SAPs [2, 10, 14, 17, 26, 37, 46]. There are equally a few recent studies in this area focusing specifically on Nigeria [3, 6, 28, 40] and others on agricultural productivity [9, 38]. These studies either examine the determinants of adoption of agricultural technologies at the household level or productivity in isolation, without delving into plot-level determinants, and productivity implications of adoption. In addition, to the best of my knowledge, there is no study examining the joint adoption of SAPs at the plot level and their effect on productivity using nationally representative, geo-referenced data in rural Nigeria. Further, there is no study examining the impact of long-term climate variability on adoption of SAPs and productivity at the plot-level in rural Nigeria.

This paper contributes to the growing literature on agricultural technology adoption, climate change and productivity in the following respect. First, this study uses nationally representative, geo-referenced plot-level data to rigorously examine the adoption of SAPs (improved seeds, organic fertilizer, inorganic fertilizer, and legume intercropping) at the plot-level in rural Nigeria. Second, this study contributes to the literature by examining the determinants of adoption and intensity of adoption of multiple SAPs at the plot-level, employing estimation strategies that allow for interrelationships between the SAPs and farmers choosing a mix of practices [4, 24, 49]. Third, this paper expands the literature on the impact of SAPs on households by examining the linkage between these technologies and crop productivity. Finally, this paper examines the impact of long-term climate variabilities on adoption and intensity thereof, and their implications for plot-level crop productivity.

The rest of the paper is structured as follows. In “Data and descriptive statistics” section, description of the data and summary statistics are presented. "Econometric framework and estimation strategy" section provides the econometric strategy and estimation procedures employed in the study. "Results and discussion" section presents the empirical results, and in "Conclusions" section conclusions and policy implications of the study are provided.

## Data and descriptive statistics

### Data

This paper uses data from the fourth wave of the Nigeria General Household Survey (GHS)—Panel, conducted by the National Bureau of Statistics (NBS), covering the 2018/2019 agricultural season [33]. The GHS-Panel is part of the Living Standards Measurement Study—Integrated Survey on Agriculture (LSMS-ISA) project of the World Bank. The LSMS team provided technical assistance, while the Bill and Melinda Gates foundation provided funding for the survey. The survey has rich information on agriculture, global positioning system (GPS), and other socioeconomic variables. The survey follows a two-stage cluster sampling procedure and is representative at the national and zonal levels, with rural/urban stratification. Given its nationally representative design, the survey was fielded in all 37 states (including the Federal Capital Territory) of Nigeria. The present study focuses on the rural sector, where crop farming is the main income generating activity of smallholder households.

The GHS-Panel 2018/2019 data contain 5025 households, of which 4976 households were successfully interviewed with complete information. Of the 4976 households, 3384 were located in rural areas, while the remaining 1592 were located in urban areas. Of the 3384 rural households, 2704 engaged in crop farming and provided complete plot-level input and output information for the 2018/2019 agricultural season. Given that one of the main objectives of this paper is examining productivity, including incorporating climate and other biophysical variables (e.g., nutrient retention, rooting conditions, salinity, etc.), the sample was restricted further to only plots that were measured using GPS device, with available coordinates. All plots cultivated and harvested by the household during the reference agriculture season and have GPS information were included in the analysis, irrespective of the crops planted on them. At the end of the cleaning process, 5616 plots from 2558 households were included in the analysis.

The GPS coordinates from the survey allow for linking household- and plot-level data to geo-referenced soil and climate variables. The publicly available GHS-Panel 2018/2019 data contain some relevant soil and weather variables. However, due to the requirements of the present study, additional rainfall and temperature variables were generated. This was done by merging the household and plot-level GPS coordinates with the Africa Rainfall Climatology version 2 (ARC2) of the National Oceanic and Atmospheric Administration’s (NOAA) database.^2^ Daily time series rainfall and temperature data were extracted for the period 1983–2017. Focusing on the planting season months (March–June), coefficient of variation of temperature, rainfall, and growing degree days (GDD) and average rainfall shortfall were generated and included as additional covariates in the adoption and intensity of adoption models. These variables also served as instruments in the production function analysis, allowing for examining the long-term impact of weather variability on agriculture technology adoption and productivity in rural Nigeria. Following Asfaw et al. [4], the rainfall shortfall variable was computed as the average distance between the yearly precipitations and their long-term mean. The computation of this variable does not include those years where the precipitation is higher than the long-term average to allow for examining long-term rainfall risk on adoption and productivity.

### Description of variables and summary statistics

#### Dependent variables

The GHS-Panel 2018/2019 has rich plot-level information on several SAPs. The SAPs considered in this study are improved seeds, inorganic fertilizer, organic fertilizer, and legume intercropping. Improved seeds include the use of modern, high yielding varieties recommended by the Federal Ministry of Agriculture and Rural Development (FMARD), adapted to different agro-ecological zones of the country. Households were asked if the seed they planted on a given plot during the agricultural season was improved, as well as the name and certification status of the seed, if improved. Thus, a plot is defined as having been planted with improved seed if the household responded yes to the question of if the seed planted is improved. The descriptive result shows that about 9% of plots were planted with improved seeds (Table 1).

**Table 1:** Definition of variables and summary statistics

Variable	Definition	Mean	Std deviation
Improved seeds	1 if improved seed was planted on plot during the 2018/19 agricultural season	0.09	0.28
Inorganic fertilizer	1 if inorganic fertilizer was used on plot during the 2018/19 agricultural season	0.43	0.49
Organic fertilizer	1 if organic fertilizer was used on plot during the 2018/19 agricultural season	0.29	0.45
Legume intercropping	1 if the plot was intercropped with a leguminous crop during the 2018/19 agricultural season	0.24	0.43
Productivity	Value of crop harvest per harvested plot area (Naira/hectare)	352,254	698,189
CV of rainfall	Coefficient of variation of rainfall during the planting season (March-June), 1983–2017	0.69	0.18
CV temperature	Coefficient of variation of daily temperature during the planting season (March-June), 1983–2017	0.05	0.02
Rainfall shortfall	Average rainfall shortfall where the annual rainfall during the growing season (March-June) is less than their long-term average (1983–2017)	19.37	7.17
CV of GDD	Coefficient of variation of growing degree days during the planting season (March-June), 1983–2017	0.22	0.21
CV days temp	Coefficient of variation of number of days where temperature is greater than 34 °C during the planting season (March-June), 1983–2017	0.82	0.98
Household size	Number of persons in the household	6.80	3.62
Dependency	Share of dependents in the household	1.12	1.17
Gender	1 if the plot manager is male	0.84	0.37
Age	Age of the plot manager in years	48.09	14.20
Education	Number of years of formal education of the plot manager	6.12	4.79
Off farm	1 if the plot manger worked in a non-farm household business	0.45	0.50
Wage work	1 if the plot manger had a wage work	0.13	0.34
Credit	1 if the household had access to credit	0.12	0.32
Remittance	1 if the household received international remittance	0.02	0.13
Extension	1 if the household had access to extension services	0.20	0.40
Plot size	Plot size planted (hectares)	0.52	0.78
Harvested plot size	Plot size harvested (hectares)	0.50	0.77
Owned land	1 if the plot was owned by the household during the 2018/19 agricultural season	0.78	0.41
Erosion	1 if there is erosion control facility on plot	0.03	0.18
Steep slope	1 if farmer perceives the plot to have a steep slope	0.22	0.41
Irrigation	1 if the plot was irrigated during the 2018/2019 agricultural season	0.03	0.18
Fertilizer price	Average price of a Kg of inorganic fertilizer (NPK or urea)	152.22	89.97
Distance	Distance from homestead to nearest market (Km)	61.99	47.72
Nutrient constraint	Nutrient availability constraint (1–5 scale, 5 = mainly water)	1.74	0.81
NDVI	Long-term average NDVI (greenness) in primary growing season (highest quarter)	0.29	0.04
Distance to household	Distance from homestead to plot (Km)	1.42	5.25
Wealth index	Wealth index, including assets, dwelling characteristics, sanitation and water	− 0.43	0.73
Agric capital index	Index of agricultural capital (implements)	0.66	1.05
TLU	Tropical livestock size of the household 12 months prior to the survey	1.54	3.48
North Central	1 if the household is located in North Central zone	0.24	0.43
North East	1 if the household is located in North East zone	0.15	0.36
North West	1 if the household is located in North West zone	0.32	0.47
South East	1 if the household is located in South East zone	0.13	0.34
South South	1 if the household is located in South South zone	0.03	0.18
South West	1 if the household is located in South West zone	0.12	0.33

In Nigeria, inorganic fertilizer comes in the form of Nitrogen, Phosphorus, Potassium (NPK), and Urea. Following Refs. [4] and [49], adoption of inorganic fertilizer is defined as 1 if the household applied NPK and or urea on the plot during the agricultural season, and 0 otherwise. Overall, households in rural Nigeria applied inorganic fertilizer to 43% of their plots (Table 1). Organic fertilizer comes in the form of animal and plant wastes, commonly manure, and or crop residues. The share of plots in the study area that received organic fertilizer is 29%. Legume intercropping involves planting a legume (e.g., groundnut) and a non-leguminous crop on the same plot during the agricultural season. Households in the survey were not asked explicitly if they used legume intercropping technology on respective plots but were asked for the crops they planted on a given plot during the season. Using this information, a variable was constructed taking the value of 1 if the household intercropped a legume crop with other crop(s) on the plot during the agricultural season, and 0 otherwise. The data show that about 24% of plots were intercropped with legumes.

In order to examine the impact of the SAPs on productivity, the dependent variable is defined as the monetary value of crop(s) per harvested plot area. For a given harvested plot, the survey asks for the value of crop harvested (excluding expected harvest). This value was then divided by the total hectares of harvested plot area and used as the measure of productivity of that plot. Productivity is measured in terms of value (Naira) per hectare instead of quantities (kilograms per hectare) because of the difficulty of aggregating different kinds of crops that may grow on the same plot and may have different productivity levels and economic values [4, 9, 38, 39]. The descriptive results show that the average yield is 352,254 Naira per hectare.^3^

#### Independent variables

Based on economic theory, the adoption and crop productivity literature [12, 24, 25, 27, 30, 37], 38, 40, 43, 49] relevant explanatory variables were included in the adoption and impact equations. The variables range from household-level demographic characteristics, credit and information access, land tenure, plot-level technical and managerial factors to climate- and soil-related variables. Table 1 provides the definitions of the right-hand side variables included in this analysis.

## Econometric framework and estimation strategy

### Conceptual framework

Rural smallholder households in Nigeria adopt a mixture of technologies on their plots, either simultaneously or sequentially. Applying multiple technologies on the same plot may result in complementarity or substitutability, where the adoption of one technology may increase the propensity to adopt another, and vice versa. With multiple SAPs, there is possible interdependence between the unobserved heterogeneity, and thus, this interdependence should be taken into account when modeling household adoption decisions involving multiple alternatives. Further, in the presence of multiple technologies, adoption becomes path dependent, where lessons learned from adopting the first technology might influence the adoption of subsequent ones.

Intensity of adoption is equally important when examining the adoption of multiple technologies at the plot level. This paper defines intensity of adoption as the number of technologies applied per plot in the growing season. Following Teklewold et al. [49], intensity of adoption is also modeled using pooled random-effects ordered probit model, given that there are multiple plots per household in some cases.

### Multivariate probit model

Following the extensive review of literature on adoption of SAPs [10, 13, 17, 18, 25, 36, 49, 50], this paper models the adoption of agricultural technologies at the plot level following the random utility framework. The decision to adopt a given technology is embedded in the general theory of random utility maximization. Households adopt improved technology or switch from traditional to an improved practice if the utility derived from the improved type is higher than that of the traditional. When farmers are face with a single technology, their decision to adoption one is not conditional on adopting another. In the case of multiple technologies, however, the interrelations (substitutability and complementarity) between the technologies should be taken into account when modeling their adoption.

Let ${U}_{j}$ denote the benefit that the *i*th household $(i=1, 2, \dots .N)$ derives from using technology $j$ $\left(j=S, F, M, L\right)$ on plot $p$ $(p=1, 2, \dots .P)$ and ${U}_{0}$ denote the benefit otherwise. Faced with $j$ technologies, the *i*th household adopts the *j*th technology on plot $p$ if ${U}_{j}>{U}_{0}$. Define the unobserved net benefit of the *i*th household using technology $j$ on plot $p$ as ${Y}_{\text{ipj}}^{*}$ which is explained by observed household- and plot-level factors (${X}_{ip}^{^{\prime}}$), climate variables (including long-term variability) $({C}_{ip}^{^{\prime}}$), and error terms (${e}_{ip}$) and specified as follows:

$${T}_{\text{ipj}}^{*}={X}_{ip}^{^{\prime}}{\beta }_{j}+{C}_{ip}^{^{\prime}}{\gamma }_{j}+{e}_{ip}.$$

Such that the binary outcome for each technology is given as

$${T}_{\text{ipj}}=\left\{\begin{array}{l} 1 \quad \forall {T}_{\text{ipj}}^{*} > 0\\ 0 \quad \forall {T}_{\text{ipj}}^{*} \le 0 \end{array}\right., \quad \left(j=S, F, M, L\right)$$

The multivariate probit (MVP) model is thus characterized by a set of binary dependent variables equal to 1 if the household adopts technology $j$ ($j$ = 1, 2, 3, 4) on plot $p$ and 0 otherwise. The error terms in the MVP model follow a multivariate normal distribution given that households can adopt multiple technologies on the same plot. The multivariate normal distribution has a zero conditional mean and variance normalized to unity to allow for identifying the parameters. The symmetric covariance matrix ($\Omega$) of the error terms in the MVP model is given by

$$\Omega =\left[\begin{array}{cccc}1& {\rho }_{sf}& {\rho }_{sm}& {\rho }_{sl}\\ {\rho }_{fs}& 1& {\rho }_{fm}& {\rho }_{fl}\\ {\rho }_{ms}& {\rho }_{mf}& 1& {\rho }_{ml}\\ {\rho }_{ls}& {\rho }_{lf}& {\rho }_{lm}& 1\end{array}\right]$$

where the non-zero off-diagonal elements represent the unobserved correlation between the error terms of the underlying technologies. Given the heteroscedastic nature of the error terms in the equations and the intra-household correlation of plot invariant covariates, the MVP model is estimated following Mundlak [31] approach by including intra-household means of plot-varying variables, such as soil quality, irrigation, slope, among others, in the model. This allows for controlling for unobserved heterogeneity.

### Ordered probit model specification

Given that the MVP technique described above is only able to identify the factors influencing the adoption of the SAPs, this paper goes a step further to estimate an ordered probit model to examine the extent to which households adopt the SAPs on their plots. Theoretically, the factors that affect the adoption of the practices may differ from the factors that determine the extent of application on plots. Generally, intensity of adoption has been measured using continuous variables (such as area planted to the technology, or the quantity of an input applied to a particular area) [1, 44, 52]. In the case of multiple technologies applied to a specific plot, it is difficult to quantify the extent of adoption in the traditional sense given that some households may adopt the full package (all four technologies), while others may use part (less than 4 technologies) on their plots [49].

Following D’Souza et al. [11], Wollni et al. [51], and Teklewold et al. [49], the present study defines intensity of adoption as the number of the SAPs applied to a specific plot during the agricultural season. While there are alternative estimators that can be used to estimate the intensity of adoption [19], the current study employs the ordered probit model due to the path-dependent nature of using multiple technologies on plot during the growing season. Plot-level intensity of SAPs adoption is defined following a latent variable model:

$${D}_{ip}^{*}={X}_{ip}^{^{\prime}}\phi +{C}_{ip}^{^{\prime}}\mu +{v}_{ip} {v}_{ip}|{X}_{ip}^{^{\prime}}, {C}_{ip}^{^{\prime}}\sim Normal\left(\mathrm{0,1}\right)$$

where ${D}_{ip}^{*}$ is a latent variable underlying the unobserved measure of the number of SAPs adopted on a given plot and ${X}_{ip}^{^{\prime}}$ and ${C}_{ip}^{^{\prime}}$ are as defined above. Given the axiom of path dependence, for a low ${D}_{ip}^{*},$ the number of SAPs is low, while the number of SAPs adopted increases as ${D}_{ip}^{*}$ gets higher. Further, let ${\alpha }_{1}<{\alpha }_{2}<{\alpha }_{3}<{\alpha }_{4}$ define unknown cut-off points or threshold parameters such that

$${D}_{ip}=\left\{ \begin{array}{*{20}l} 0 & \quad \forall {D}_{ip}^{*}\le {\alpha }_{1} \\ 1 & \quad \forall {\alpha }_{1}<{D}_{ip}^{*}\le {\alpha }_{2}\\ 2 & \quad \forall {\alpha }_{2}<{D}_{ip}^{*}\le {\alpha }_{3}\\ 3 & \quad \forall {\alpha }_{3}<{D}_{ip}^{*}\le {\alpha }_{4}\\ 4 & \quad \forall {D}_{ip}^{*}>{\alpha }_{4}\end{array}\right. .$$

This equation is estimated using the Maximum Likelihood estimation procedure.

### Estimating the impact on productivity

The paper further examines the impact of SAPs on crop productivity using the production function approach. Crop productivity is measured as the value of total harvests per area harvested of plot (Naira/hectare) [4, 5, 23]. The impact of the ${j}^{th}$ technology on plot-level productivity ($\psi$) is modeled by estimating the following equation:

$${Q}_{p}={T}_{ip}^{\mathrm{^{\prime}}}\psi + {X}_{ip}^{\mathrm{^{\prime}}}\varphi +{C}_{ip}^{^{\prime}}\lambda +{\varepsilon }_{ip,}$$

where ${Q}_{p}$ is the logarithm of the value of output per hectare from plot $p$, and ${T}_{ip}^{\mathrm{^{\prime}}}$ is a vector of SAPs. It is obvious that ${T}_{ip}^{\mathrm{^{\prime}}}$ is endogenous – differences in factors (household, plot, and climate) that affect the choice of technologies might also influence productivity (non-zero correlation between ${T}_{ip}^{\mathrm{^{\prime}}}$ and ${\varepsilon }_{ip}$). Thus, the choice of technologies is endogenously, rather than exogenously, determined in the production function. This gives rise to multiple endogeneity problems given the number of SAPs. The implication is that estimating the equation without taking into account these endogeneity problems may lead to bias estimates and misleading conclusions.

This paper employed the instrumental variables (IV) procedure proposed by Lewbel [29] (*ivreg2h* program in Stata by Baum and Schaffer [7] in Stata) to correct for the endogeneity problems. The *ivreg2h* procedure works by using the model’s data (exogenous variables) to generate additional instruments to instrument the potential endogenous variables. These model generated instruments are used alongside the externally supplied instruments to allow for achieving identification and also correct for possible weak instruments.

Like in every IV technique, the choice of external instruments is critical for the *ivreg2h* procedure, as the consistency of the IV procedure lies in the validity of instruments. Theoretically, the choice of instruments should satisfy two conditions. First, the instruments should be correlated with the endogenous variables in the production model. Second, they should be uncorrelated with the unobserved variables (error term) that may affect productivity. Following economic theory and empirical literature [4, 9], long-term variability in climate during the planting season (March–June) over the period 1983–2017 were used as instruments. Specifically, rainfall shortfall, coefficients of variation of growing degree days (GDD), rainfall, daily temperature, and number of days that the maximum temperature is greater than 34 degree Celsius were used as instruments. While at levels these climate variables might be correlated with productivity (e.g., high rainfall results in increased production, high temperature results in low yields, etc.), using the long-term variability in these covariates potentially generates uncertainty about productivity [4]. The validity of these instruments was tested, and the results are presented in subsequent sections.

## Results and discussion

### Joint, marginal, conditional, and unconditional adoption probabilities

Table 2 presents the joint and marginal probabilities of adoption of SAPs in rural Nigeria. This table shows that, among the SAPs under consideration, inorganic fertilizer is the most used SAP. Specifically, inorganic fertilizer is used as a single technology on about 18.56% of plots, followed by organic fertilizer (4.95%) and legume intercropping (4.74%). Inorganic fertilizer was adopted jointly with improved seeds on 1.75% of plots and in combination with organic fertilizer, 8.5% of plots, and jointly with organic fertilizer and legume intercropping, 7.38% of plots. Improved seeds were used as a single technology on 3.64% of plots, in combination with organic fertilizer, 0.76% of plots, while jointly with organic and inorganic fertilizer, 1.2% of plots.

**Table 2:** Joint and marginal probabilities of SAPs adoption (%)

Percent adopting in	Joint probability	Marginal probabilities			
		Improved Seeds	Inorganic fertilizer	Organic fertilizer	Legume Intercropping
Improved seed only	3.64	3.64			
Inorganic fertilizer only	18.56		18.56		
Organic fertilizer only	4.95			4.95	
Intercropping only	4.74				4.74
Improved seed and inorganic fertilizer	1.75	1.75	1.75		
Improved seed and organic fertilizer	0.76	0.76		0.76	
Improved seed and intercropping	0.20	0.20			0.20
Inorganic fertilizer and organic fertilizer	8.50		8.50	8.50	
Inorganic fertilizer and intercropping	4.34		4.34		4.34
Organic fertilizer and intercropping	5.87			5.87	5.87
Improved seed, inorganic fertilizer, organic fertilizer	1.20	1.20	1.20	1.20	
Improved seed, inorganic fertilizer, intercropping	0.65	0.65	0.65		0.65
Improved seed, organic fertilizer, intercropping	0.16	0.16		0.16	0.16
Inorganic fertilizer, organic fertilizer, intercropping	7.38		7.38	7.38	7.38
All four	0.39	0.39	0.39	0.39	0.39
None (plot did not receive any of the technologies)	36.91				
Total	100.00	9.33	36.06	24.41	19.71

In order to explore descriptively, the interdependence among SAPs adoption, the conditional and unconditional probabilities of adoption were estimated (Table 3). Unconditionally, 9% of plots received improved seeds, while inorganic fertilizer and legume intercropping were applied to 43% and 24% of plots, respectively. The adoption probability of inorganic fertilizers, however, decreases from 43 to 41% if the plot received organic fertilizer, but increases to 74% if the plot received improved seeds and legume intercropping. The results show substitutability between organic and inorganic fertilizer adoption, as the probability of a plot receiving inorganic fertilizer decreases if the plot received organic fertilizer. Further, the adoption probability of improved seeds increases from 9 to 46% if the plot received inorganic fertilizers, but reduces to 4% if the plot received organic fertilizer and legume intercropping. This suggests that in the presence of multiple technologies, more needs to be done to nudge farmers to adopt improved seeds on their plots.

**Table 3:** Conditional and unconditional adoption probabilities of SAPs

Condition	Improved Seeds	Inorganic fertilizer	Organic fertilizer	Legume Intercropping
$P({Y}_{j}=1)$	0.09	0.43	0.29	0.24
$P\left({Y}_{j}=1\right| {Y}_{s}=1)$	1	0.09	0.09	0.06
$P\left({Y}_{j}=1\right| {Y}_{F}=1)$	0.46	1	0.60	0.54
$P\left({Y}_{j}=1\right| {Y}_{M}=1)$	0.29	0.41	1	0.58
$P\left({Y}_{j}=1\right| {Y}_{L}=1)$	0.16	0.30	0.47	1
$P\left({Y}_{j}=1\right| {Y}_{s}=1, {Y}_{F}=1)$	1	1	0.40	0.26
$P\left({Y}_{j}=1\right| {Y}_{s}=1, {Y}_{M}=1)$	1	0.63	1	0.22
$P\left({Y}_{j}=1\right| {Y}_{s}=1, {Y}_{L}=1)$	1	0.74	0.39	1
$P\left({Y}_{j}=1\right| {Y}_{F}=1, {Y}_{M}=1)$	0.09	1	1	0.44
$P\left({Y}_{j}=1\right| {Y}_{F}=1, {Y}_{L}=1)$	0.08	1	0.61	1
$P\left({Y}_{j}=1\right| {Y}_{M}=1, {Y}_{L}=1)$	0.04	0.56	1	1
$P\left({Y}_{j}=1\right| {Y}_{S}=1, {Y}_{F}=1, {Y}_{M}=1)$	1	1	1	0.24
$P\left({Y}_{j}=1\right| {Y}_{S}=1, {Y}_{F}=1, {Y}_{L}=1)$	1	1	0.37	1
$P\left({Y}_{j}=1\right| {Y}_{S}=1, {Y}_{M}=1, {Y}_{L}=1)$	1	0.70	1	1
$P\left({Y}_{j}=1\right| {Y}_{F}=1, {Y}_{M}=1, {Y}_{L}=1)$	0.05	1.00	1	1

### Determinants of plot-level SAPs adoption: MVP results

The maximum likelihood estimates of the MVP model of the factors affecting the adoption of SAPs at the plot-level in rural Nigeria are presented in Tables 4 and 5. The model fits the data reasonably well, as the Wald test ${(\chi }^{2}\left(156\right)= 4083.32, P=0.000)$ of the hypothesis that all regression coefficients in each equation are jointly equal to zero is rejected. Similarly, the likelihood ratio test of the hypothesis that the covariance of the error terms across equations are not correlated is also rejected ${(\chi }^{2}\left(6\right)=51.58, P=0.000)$, warranting the MVP model instead of separate single-equation probit models.

**Table 4:** Determinants of SAPs adoption—multivariate probit model

Variable	Improved seeds		Inorganic fertilizer		Organic fertilizer		Legume intercropping	
	Coefficient	Std dev	Coefficient	Std dev	Coefficient	Std dev	Coefficient	Std dev
Log (CV rainfall)	− 0.732**	0.335	0.186	0.259	0.624**	0.311	− 0.388	0.333
Log (CV temperature)	0.355	0.228	1.423***	0.158	0.742***	0.199	− 0.324	0.198
Rainfall shortfall	− 0.029***	0.008	0.008	0.007	− 0.003	0.008	− 0.036***	0.009
Log (CV of GDD)	0.004	0.082	− 0.469***	0.061	− 0.198***	0.064	0.014	0.066
Log (CV days temp)	0.352***	0.063	− 0.089	0.057	− 0.516***	0.067	− 0.430***	0.068
Household size	− 0.002	0.008	− 0.001	0.006	− 0.009	0.006	− 0.001	0.007
Dependency	0.036*	0.020	− 0.013	0.017	0.015	0.019	− 0.029	0.021
Gender	− 0.116	0.071	0.061	0.058	0.189***	0.068	− 0.250***	0.074
Age	− 0.001	0.002	0.002	0.001	0.002	0.002	0.002	0.002
Education	0.011*	0.006	0.015***	0.005	− 0.008	0.005	− 0.005	0.005
Off farm	0.206***	0.054	0.129***	0.040	0.125***	0.045	0.037	0.046
Wage work	0.247***	0.077	0.112*	0.059	− 0.142*	0.075	0.152**	0.069
Credit	− 0.141*	0.082	− 0.081	0.059	0.156**	0.063	0.042	0.072
Remittance	0.375**	0.151	− 0.092	0.154	− 0.075	0.175	− 0.071	0.209
Extension	0.201***	0.064	0.165***	0.048	− 0.065	0.054	− 0.062	0.055
Plot size	0.025	0.046	0.104***	0.033	0.080**	0.039	0.312***	0.038
Owned land	0.075	0.127	− 0.040	0.098	0.069	0.114	0.206*	0.114
Erosion	− 0.058	0.324	− 0.091	0.233	− 0.029	0.287	− 0.104	0.260
Steep slope	0.093	0.122	− 0.020	0.082	− 0.039	0.093	− 0.092	0.089
Irrigation	− 0.120	0.258	0.252	0.198	− 0.346	0.215	− 0.988***	0.222
Fertilizer price	0.000	0.000	0.000	0.000	− 0.001**	0.000	− 0.000	0.000
Distance	0.002***	0.001	− 0.003***	0.000	− 0.003***	0.000	− 0.000	0.000
Nutrient constraint	0.251***	0.042	− 0.016	0.033	0.055	0.038	0.105**	0.042
NDVI	1.305*	0.671	− 6.233***	0.675	− 3.188***	0.745	− 2.425**	0.965
Distance to household	0.001	0.026	-0.007	0.013	− 0.013	0.016	− 0.024**	0.012
Wealth index	0.163***	0.040	0.194***	0.034	0.151***	0.038	0.000	0.040
Agric capital index	− 0.105***	0.029	0.024	0.020	0.111***	0.021	− 0.036	0.022
TLU	0.015**	0.006	0.010*	0.005	0.032***	0.006	0.000	0.006
North Central	− 0.114	0.236	0.645***	0.227	− 0.068	0.303	0.861***	0.324
North East	0.921***	0.269	1.166***	0.237	0.043	0.315	0.936***	0.330
North West	1.120***	0.290	1.479***	0.248	0.391	0.322	1.034***	0.341
South East	− 0.022	0.226	1.085***	0.234	0.881***	0.284	0.385	0.349
South West	0.656***	0.217	− 0.373	0.255	0.349	0.293	0.254	0.369
Constant	− 1.351	0.823	3.667***	0.600	1.106	0.748	− 1.514**	0.756
Joint significance of mean of plot-varying covariates [χ2 (24)]					76.48***			
Sample size					5,616			
Wald (156)					4083.32***			

* , **, and *** indicate statistical significance at 10%, 5%, and 1%, respectively

**Table 5:** Estimated covariance matrix of the multivariate probit model regression between SAPs

	${\rho }_{S}$	${\rho }_{F}$	${\rho }_{M}$	${\rho }_{L}$
${\rho }_{S}$	1			
${\rho }_{F}$	0.071*	1		
${\rho }_{M}$	0.039	0.074***	1	
${\rho }_{L}$	− 0.026	− 0.009	0.192***	1
Likelihood ratio test of:${\rho }_{SF}= {\rho }_{SM} = {\rho }_{SL}= {\rho }_{FM} ={\rho }_{FL} ={\rho }_{ML}= 0$ ${(\chi }^{2}\left(6\right)= 51.58, P=0.000)$				

*, ** and *** indicate statistical significance at 10%, 5% and 1%, respectively

*S* seed, *F* = inorganic fertilizer, *M* organic fertilizer, *L* legume intercropping

The correlation matrix in Table 5 shows that the estimated correlations between the SAPs is significantly different from zero, mostly showing positive relationships. These indicate that the probability of a plot receiving a particular technology is conditional on the propensity of that same plot receiving any other SAP, again supporting the use of MVP. The results further indicate that the use of inorganic fertilizer is complementary to the use of improved seeds, as is the use of organic fertilizer and legume intercropping.

The MVP model results vary substantially across the SAPs equations, indicating the heterogeneous nature of the results, and therefore warrant discussing the results separately. The results presented in Table 4 follows Mundlak’s [31] approach where means of plot-varying covariates were included in the MVP estimation to control for possible unobserved heterogeneity (correlation between unobservable plot-level invariant factors and the decision to adopt the technologies). The Wald test result ${(\chi }^{2}\left(24\right)= 76.48, P=0.000)$ indicates correlation between plot-varying covariates and the households’ decision to SAPs on plot.

The analysis reveals the importance of long-term climate variabilities on households’ decision to adopt the SAPs. Households located in areas with high long-term variability in rainfall are less likely to use improved seeds, but more likely to adopt organic fertilizers. Further, in areas with high variability in daily temperatures, households are more likely to use organic and inorganic fertilizers to circumvent potential negative impact of temperatures during the growing season. Similarly, high variations in growing degree days (GDD) decrease the propensity of a plot receiving organic and inorganic fertilizers. In addition, high long-term variations in the number of days that the average maximum temperatures are above 34 °C during the planting season (March–June) deters farmers from using organic fertilizers and legume intercropping, while at the same time spurring the use of improved seeds. Finally, rainfall shortfall during the planting season tends to discourage the use of improved seeds and legume intercropping.

The results demonstrate further the importance of household demographic characteristics on SAPs adoption in rural Nigeria. The number of years of education of the plot manager has a positive significant effect on inorganic fertilizer and improved seeds adoption but no significant effect on the use of the other technologies. Male-managed plots are more likely to receive organic fertilizers compared to female-managed plots. The availability of road infrastructure, proxied by distance from the household’s dwelling to main market, negatively affect the use of most of the technologies. Specifically, households residing farther from the main markets are less likely to use inorganic fertilizers on their plot, which is expected given the probable high transaction cost of obtaining the inputs.

Households with access to extension services are more likely to adopt improved seeds and inorganic fertilizers, indicating the importance of agricultural extension services on SAPs adoption in rural Nigeria, corroborating the findings of Tambo and Abdoulaye [48]. The results also show the importance of household’s access to non-agricultural sources of income on adopting the underlying technologies. For instance, households who received international remittances are more likely to adopt improved seeds than those otherwise. Similarly, plots where the manager is engaged in non-farm business are more likely to received improved seeds, organic, and inorganic fertilizers. Wage-earning plot managers are more likely to apply improved seeds and inorganic fertilizers to their plots, but less likely to adopt organic fertilizers.

Plot size has a positive significant effect on organic and inorganic fertilizers adoption, as well as the propensity of a plot being intercropped with legumes. This result is consistent with the findings of Kassie et al. [23] for improved groundnut varieties adoption in Uganda. In addition, households tend to intercrop plots that they own with legumes, given that the full benefit of leguminous crops (nitrogen fixing) might not be realized until subsequent agricultural seasons.

The long-term greenness of the location of the household, proxied by the normalized difference vegetation index (NDVI), decreases the probability of farmers applying organic and inorganic fertilizers to their plots. Agronomically, the greenness of a vegetation denotes fertility, and therefore farmers may not be enthused about investing in soil enhancing technologies if the greenness index of the area is high. The nutrient availability constraints results show that households that face higher constraints in terms of nutrient availability are more likely to utilize improved seeds and legume intercropping.

The use of SAPs is also explained by household wealth and agricultural capital indices. Households with high wealth index use technologies that require initial capital investment, such as improved seeds, organic, and inorganic fertilizers. This finding is consistent with Tekelwold et al. [49] and Asfaw et al. [4] that found wealth to positively explain household adoption of their underlying technologies. As expected, the use of organic fertilizer is positively explained by livestock ownership, consistent with Kassie et al. [24] and Asfaw et al. [4]. While agricultural capital index has a negative effect on the use of improved seeds, it tends to facilitate the use of organic fertilizers.

### Intensity of adoption—ordered probit model results

The ordered probit model results (both pooled and random effects) of the intensity of SAPs adoption are presented in Table 6. The estimation was done using Mundlak [31] approach by including mean of plot-varying covariates in the model estimation.^4^ The approach allows for examining the marginal impact of each covariate on the degree of adoption (number of technologies adopted per plot) separately. The Chi-squared statistic of the joint significance of all coefficients in the model rejects the null hypothesis at the 1% significance level ${(\chi }^{2}\left(39\right)= 2546.51, P=0.000)$, indicating that the covariates jointly explain the intensity of adoption of SAPs. Given the multiple plots per household, a random-effects ordered probit model was also estimated for robustness check (see the last two columns of Table 6).^5^

**Table 6:** Determinants of intensity of adoption – ordered probit model

Variable	Pooled ordered probit							Random effects ordered probit	
	Coef	Std error	Marginal effects						
			Prob (D = 0 | X)	Prob (D = 1 | X)	Prob (D = 2 | X)	Prob (D = 3 | X)	Prob (D = 4 | X)	Coef	Std error
Log (CV rainfall)	0.260	0.222	− 0.097	0.021	0.059	0.017	0.0002	0.203	0.390
Log (CV temperature)	0.869***	0.126	− 0.323***	0.070***	0.196***	0.056***	0.0007***	1.075***	0.222
Rainfall shortfall	− 0.021***	0.006	0.008***	− 0.002***	-0.005***	− 0.001***	0.0000**	− 0.018*	0.011
Log (CV GDD)	− 0.256***	0.049	0.095***	− 0.021***	− 0.058***	− 0.017***	− 0.0002***	− 0.371***	0.089
Log (CV days temp)	− 0.280***	0.045	0.104***	− 0.022***	− 0.063***	− 0.018***	− 0.0002***	− 0.487***	0.081
Household size	− 0.005	0.005	0.002	0.000	− 0.001	0.000	0.0000	− 0.003	0.008
Dependency	− 0.003	0.015	0.001	0.000	− 0.001	0.000	0.0000	0.001	0.028
Gender	0.033	0.047	− 0.012	0.003	0.007	0.002	0.0000	− 0.012	0.077
Age	0.002*	0.001	− 0.001*	0.000*	0.000*	0.000*	0.0000	0.003	0.002
Education	0.006	0.004	− 0.002	0.000	0.001	0.000	0.0000	0.011	0.007
Off farm	0.187***	0.033	− 0.069***	0.014***	0.042***	0.012***	0.0002***	0.256***	0.062
Wage work	0.105**	0.050	− 0.038**	0.007***	0.024**	0.007**	0.0001	0.187**	0.088
Credit	− 0.021	0.047	0.008	− 0.002	− 0.005	− 0.001	0.0000	− 0.046	0.089
Remittance	0.081	0.124	− 0.030	0.005	0.019	0.006	0.0001	0.121	0.225
Extension	0.057	0.041	− 0.021	0.004	0.013	0.004	0.0001	0.101	0.074
Plot size	0.192***	0.027	− 0.071***	0.015***	0.043***	0.012***	0.0002***	0.276***	0.031
Owned land	0.086	0.082	− 0.032	0.008	0.019	0.005	0.0001	0.127	0.082
Erosion	− 0.083	0.192	0.031	− 0.008	− 0.018	− 0.005	− 0.0001	− 0.095	0.210
Steep slope	− 0.021	0.066	0.008	− 0.002	− 0.005	− 0.001	0.0000	− 0.029	0.079
Irrigation	− 0.392**	0.168	0.152**	− 0.056*	− 0.079***	− 0.018***	− 0.0002**	− 0.539**	0.225
Fertilizer price	0.000	0.000	0.000	0.000	0.000	0.000	0.0000	0.000	0.000
Distance	− 0.002***	0.000	0.001***	0.000***	0.000***	0.000***	0.0000***	− 0.002***	0.001
Nutrient constraint	0.128***	0.028	− 0.048***	0.010***	0.029***	0.008***	0.0001***	0.174***	0.049
NDVI	− 4.976***	0.531	1.848***	− 0.399***	− 1.123***	− 0.322***	− 0.0043***	− 7.633***	0.966
Distance to household	− 0.014*	0.008	0.005**	− 0.001*	− 0.003*	− 0.001*	0.0000	− 0.020*	0.013
Wealth index	0.183***	0.028	− 0.068***	0.015***	0.041***	0.012***	0.0002***	0.235***	0.051
Agric capital index	0.024	0.016	− 0.009	0.002	0.005	0.002	0.0000	0.048	0.030
TLU	0.020***	0.005	− 0.008***	0.002***	0.005***	0.001***	0.0000**	0.028***	0.009
North Central	0.340**	0.159	− 0.121**	0.014***	0.079**	0.027*	0.000	0.519**	0.251
North East	0.853***	0.168	− 0.283***	0.004	0.195***	0.082***	0.002*	1.274***	0.272
North West	1.233***	0.180	− 0.373***	− 0.046*	0.265***	0.149***	0.005**	1.752***	0.297
South East	0.851***	0.164	− 0.268***	− 0.024	0.195***	0.094***	0.002*	0.991***	0.257
South West	0.499***	0.168		0.001	0.118***	0.047**	0.001	0.593**	0.265
Log likelihood		− 5825.19						− 5479.23	
Wald [χ2 (39)]		2546.51***	− 0.167***					1130.60***	
Joint significance of mean of plot-varying covariates [χ2 (6)]		53.45***						53.74***	
${\alpha }_{1}$	− 3.14	0.481						− 3.756	0.840
${\alpha }_{2}$	− 1.970	0.480						− 2.087	0.838
${\alpha }_{3}$	− 0.863	0.479						− 0.541	0.839
${\alpha }_{4}$	0.742	0.487						1.710	0.852

*, ** and *** indicate statistical significance at 10%, 5% and 1%, respectively

The results reveal the importance of climate variables on adoption intensity. Households located in areas with high long-term rainfall shortfall during the growing season are less likely to apply two technologies, but more likely to not use any technology. High variability in daily temperatures tend to foster adoption intensity. However, high variability in growing degree days and the number of days where the maximum temperature is above 34 degree Celsius during the growing season tend to decrease the intensity of use of the technologies on plot.

The results also show that involvement in non-farm activities increase the intensity of adoption of the technologies. Specifically, farmers who work in a non-farm business are about 6.8% more likely to adopt at least one technology and 1.2% more likely to adopt more than two technologies on their plot.^6^ Similarly, wage work also enhances the intensive use of the technologies; plots whose managers have wage income are about 3.8% more likely to receive at least one technology, compared with those whose managers have no wage income.

Road infrastructure, proxied by distance from household’s dwelling to main market, has a negative significant effect on the intensity of adoption. Households who are farther from the main market are about 0.01% less likely to adopt more than one technology and 0.1% more likely to not use any SAP. Similarly, distance from plot to homestead has negative significant effect on adoption intensity, corroborating Teklewold et al. [49] and Oyetunde-Usman [40] who found negative relationship between plot distance and intensity of adoption.

Another important geographic variable that has negative significant effect on the intensity of adoption is greenness index. Households located in areas with high long-term greenness index are less likely to adopt at least one technology. Households located in the north-west part of the country are less likely to adopt at least one technology on their plots, while those located in the north central zone are more likely to do so. In fact, rural households from the north-east are about 27.9% more likely to adopt at least 2 technologies. Household wealth index and livestock size also positively influence the intensity of use of the technologies at the plot level.

### Impact of technology adoption on productivity

In Table 7, the OLS and *ivreg2h* results of the impact of SAPs on crop productivity are presented. The OLS estimator provides the impact of the SAPs on crop productivity, without considering potential endogeneity problems of the SAPs in the productivity model. The OLS estimator assumes that the use of these technologies is exogenously determined within the production function. Endogeneity, however, occurs when there is a non-zero correlation between the error term of the production function and other covariates. For instance, there might be unobserved variables that affect crop productivity and also determines the adoption of the technologies. The endogeneity test result suggests that the SAPs are endogenous in the production function.^7^ Thus, using the results from the OLS to explain the impact of SAPs on crop productivity will be bias, resulting in misleading conclusion and recommendations. To surmount this, the *ivreg2h* technique is employed to correct for the shortcomings of the OLS.

**Table 7:** Impact of SAPs on plot-level productivity (log value of harvest per hectare)

Variable	OLS		IVREG2H	
	Coefficient	Std error	Coefficient	Std error
Improved seeds	0.032	0.055	0.104	0.146
Inorganic fertilizer	0.324***	0.034	0.305*	0.179
Organic fertilizer	0.019	0.040	− 0.427***	0.140
Legume intercropping	− 0.092**	0.038	0.034	0.097
Growing season rainfall (mm)	0.000*	0.000	0.000	0.000
Start of wettest dekad in 2018	− 0.017**	0.008	− 0.019*	0.010
Household size	0.027	0.029	0.047	0.034
Dependency ratio	0.043***	0.014	0.042***	0.016
Gender	− 0.001	0.001	− 0.001	0.001
Age	0.156***	0.051	0.161***	0.056
Education	− 0.004	0.004	− 0.007*	0.005
Off farm	− 0.044	0.033	− 0.031	0.041
Wage work	− 0.104**	0.053	− 0.109*	0.063
Extension	0.093**	0.042	0.105**	0.048
Own plot	0.032	0.039	0.055	0.043
Erosion control	− 0.132	0.139	− 0.215	0.139
Steep slope	− 0.033	0.038	− 0.039	0.041
Irrigation	0.244***	0.094	0.268***	0.099
Distance to market	0.000	0.000	− 0.001	0.000
Nutrient constraint	− 0.148***	0.025	− 0.134***	0.028
NDVI in 2018	1.805***	0.406	1.635***	0.561
Distance to household	0.002	0.004	0.002	0.004
Wealth Index	0.078***	0.030	0.093***	0.036
Agric capital index	0.019	0.015	0.030	0.019
Tropical livestock unit	− 0.010***	0.004	− 0.008	0.005
North Central	0.011	0.139	− 0.028	0.162
North East	− 0.398***	0.140	− 0.409**	0.170
North West	0.040	0.144	0.156	0.195
South East	0.278*	0.156	0.268	0.183
South West	0.613***	0.164	0.545***	0.194
Constant	11.856***	0.283	11.966***	0.374
Sample size	5,616		5616	
R^2^	0.070		0.050	
Wald	15.8***		10.54***	
Underidentification test			136.19**	
Weakidentification test			2.673***	
Overidentification test (Hansen J statistic)			56.60	

*, ** and *** indicate statistical significance at 10, 5 and 1%, respectively

The *ivreg2h* is a Stata program contributed by Baum and Schaffer [7]. The program allows for estimating instrumental variables regression with an option to generate instruments using Lewbel [29] method to control for potential endogeneity problems. This technique also allows for the identification of structural parameters in regression models with endogenous or mismeasured regressors in the absence of traditional identifying information, such as external instruments or repeated measurements.

This approach of Lewbel's allows for constructing instruments as simple functions of the model's data (exogenous variables). For each regressor, *ivreg2h* creates standard form (centered) variables and used as instruments. These standard, model generated instruments can either by themselves serve to instrument the endogenous variables or can be combined with the external instruments (in this case the long-term climate variables presented earlier). Like other IV estimators, the validity of these instruments is verified in the *ivreg2h* by conducting three tests—underidentification, weakidentification, and overidentification, presented at the bottom of Table 7. Further on, the discussion focuses on the results from the *ivreg2h* estimation procedure. While the test results confirm the validity of the chosen instruments, it is important to emphasize that no instrumental variable approach is perfect. Thus, while the results presented below are vital, they should be interpreted and applied with caution.

The results indicate that the adoption of improved seeds has no significant effect on plot-level productivity, though it has the expected sign. As expected, however, the use of inorganic fertilizers significantly increases crop productivity. The use of organic fertilizer, on the other hand, makes negative significant contribution to productivity. This can possibly be attributed to the fact that some organic fertilizers, especially crop residues and animal droppings, take longer to decompose and render their benefit within the short growing season, and the strong positive correlation between inorganic and organic fertilizer discussed earlier under the conditional probabilities. This finding is also consistent with that of Asfaw et al. [4] who found negative correlation between crop residues and crop productivity in Niger. The adoption of legume-cereal intercropping also shows no significant effect on crop productivity. One possible explanation for this result is that households might be planting several crops on the same plot, more than the soil fertility capacity of the plot can handle. Moreover, the benefit of adopting legume-cereal intercropping technology may not accrue to the soil within the same growing season.

This paper looks further at how crop productivity is explained by household and plot-level factors. As expected, the results show that high greenness (NDVI) during the growing season increases agricultural productivity. Delay in the start of the wettest dekad is negatively correlated with productivity. We also see that the amount of rainfall during the growing season positively influences productivity. Soil characteristics of the plot, such as topography, nutrient availability, and water retentions, are critical in explaining plot productivity. The results show that plots with high nutrient availability constraints are less productive. Erosion is generally more prevalent on steep plots, potentially washing away the topsoil and rendering them less productive. As expected, irrigation and access to agricultural extension services positively impacts productivity.

Crop productivity from plots operated by older farmers tend to be significantly higher than those managed by younger farmers. Moreover, plots operated by households with high dependency ratios are more productive, compared to those otherwise. As expected, crop productivity tends to increase with household wealth. Plot-level productivity also varies inversely with the size of livestock (TLU) owned by the household; households with large livestock sizes tend to be less productive. Large TLU might require households reallocating their resources from crop to livestock production, potentially leading to low crop output.

## Conclusions

Sustainable agricultural practices have long been hailed as positive production function shifters, and empirical evidence indicates that the adoption of these practices depends on a number of household characteristics, plot-level technical factors, as well as weather variables. This study aimed to unravel the factors that influence the adoption and intensity thereof of multiple SAPs, and their impact on crop productivity, using nationally representative plot-level data from rural Nigeria. The multivariate and ordered probit models were used to identify the determinants of adoption and intensity of use, respectively. The impact of adoption on crop productivity was examined using the instrumental variables approach to control for endogeneity and achieve identification.

The results indicate that the application of organic and inorganic fertilizer to plots are negatively and positively correlated with crop productivity, respectively. This study also finds the importance of soil fertility and climate-related variables on crop productivity. The results reveal complementarities and substitutabilities of SAPs use at the plot-level, implying that policies geared at promoting the adoption of the SAPs should take into account these interdependencies.

Despite these interdependencies, the factors that influence the adoption and intensity of use of the SAPs are heterogeneous: household demographic characteristics, plot-level technical factors, and long-term climate variabilities. High long-term rainfall variability tends to discourage farmers from using organic fertilizers and improved seeds. Similarly, high variability in temperatures tends to nudge farmers to use organic, inorganic, and legume intercropping, and their combinations on their plots. These underscore the importance of favorable long-term climatic conditions to households’ adoption decisions of SAPs.

Plots, whose managers had access to extension services, have spent more years in school, have off-farm income generating activities and live in households with high wealth index, are more likely to receive improved seeds and inorganic fertilizers. Similarly, the use of organic fertilizer is stimulated by gender of the plot manager, long-term greenness (negatively), distance to market, and wealth indices.

These results have implications for rural development policies in Nigeria aimed at nudging farmers to adopt multiple technologies, while enhancing an outward shift of their crop production functions. First, given the strong correlation between extension access and adoption of SAPs, it is important that agricultural extension service providers in rural Nigeria be well resourced technically and financially so they can reach smallholder households in rural areas with the knowledge and benefits of these practices. In addition, smallholder households should be encouraged to diversify their livelihood portfolios to include non-farm income generating activities. Further, given the strong relationship between long-term climate variabilities and SAPs adoption and productivity, it is important that agricultural Research and Development target factors that respond to climate variabilities *(such as drought resistant and early maturing varieties). There is also a need for improved* infrastructures (road networks to allow easy market access as well as access to credit) to allow farmers access these innovations.

Finally, the adoption of improved agricultural practices has potential to impact household welfare beyond plot-level productivity. Thus, future studies need to examine the welfare (food security, consumption, dietary diversity) implications of adopting multiple agricultural technologies in rural Nigeria.

## Data Availability

The data used for the analysis are available on the Nigeria NBS and the World Bank’s microdata libraries.

## Footnotes

1 Using September 2019 exchange rate of 360 Naira to USD1.00 from www.oanda.com.

2 https://catalog.data.gov/dataset/climate-prediction-center-cpc-africa-rainfall-climatology-version-2-0-arc2.

3 This is equivalent to USD 2600 using 2019 Purchasing Power Parity of 135.39.

4 The joint test of the mean of plot-varying covariates is significantly different from zero, implying a correlation between observed and unobserved heterogeneity, thus justifying the use of Mundlak’s procedure.

5 Likelihood ratio test was conducted to establish the plausibility of estimating a random effects model, where the null hypothesis is that the correlation between two successive errors terms of plots belonging to the same household is zero. The test rejects the null hypothesis at the 1% level ${(\chi }^{2}\left(6\right)= 53.45, p=0.00)$, justifying the estimation of a random effects ordered probit model.

6 Note that for the ordered probit, the magnitudes were computed by summing up the marginal effects (ME) of the respective intensities. For instance, the probability of adopting only 1 practice = ME1; probability of adopting 2 practices = ME1 + ME2; probability of adopting more than 2 practices = ME3 + ME4 + ME5; probability of adopting 2 or more practices = ME2 + ME3 + ME4 + ME5; and probability of not adopting any practice = ME0.

7 The *ivreg2h* provides a C statistic that tests endogeneity of the included instruments. The C statistics is defined as the difference of the Sargan-Hansen statistic of the equation with the smaller set of instruments (valid under both the null and alternative hypotheses) and the equation with the full set of instruments, i.e., including the instruments whose validity is suspect. Under the null hypothesis that both the smaller set of instruments and the additional, suspect instruments are valid, the C statistic is distributed as chi-squared in the number of instruments tested. The C statistics ${(\chi }^{2}=13.002 p=0.0234)$ rejects the null hypothesis, indicating that the technology variables are endogenous in the productivity model.
